# Cardiovascular-Kidney-Metabolic Syndrome: Is the Evidence Keeping Pace with the Concept?

**DOI:** 10.1159/crm/abtag012

**Published:** 2026-09-18

**Authors:** Amir Kazory, Abhilash Koratala, Claudio Ronco

**Affiliations:** a Division of Nephrology, Hypertension, and Renal Transplantation, University of Florida, Gainesville, FL, USA; b Medical College of Wisconsin, Milwaukee, WI, USA; c Department of Nephrology, San Bortolo Hospital and International Renal Research Institute of Vicenza, Vicenza, Italy; d Department of Medicine, University of Padova, Padova, Italy

**Keywords:** Cardiovascular-kidney-metabolic syndrome, Cardiovascular diseases, Kidney disease, Diabetes, Obesity

## Background

Few clinicians would dispute that cardiovascular disease, chronic kidney disease (CKD), obesity, and diabetes are closely interconnected and would be best managed through an integrated, multidisciplinary approach. Indeed, one of the greatest accomplishments of the cardiovascular-kidney-metabolic (CKM) initiative, presented by the American Heart Association (AHA) in 2023, has been to encourage collaboration across traditionally separate specialties while emphasizing shared risk factors and opportunities for prevention. The recent publication of the first multi-society clinical guideline for CKM syndrome further underscores the momentum behind this initiative and the commitment of major professional societies to integrated cardiorenal-metabolic care [1].

We recently argued that the momentum behind CKM had begun to outpace the evidence supporting it as a distinct clinical syndrome rather than a useful organizational framework [2]. Since then, an expanding body of observational studies has attempted to validate CKM staging in diverse populations, from large biobank cohorts to nationally representative surveys. Whereas our earlier article was largely conceptual, we now use this newer evidence to test those concerns directly. Overall, the literature has sharpened rather than resolved the concerns we previously raised. What remains uncertain is not whether cardiovascular, kidney, and metabolic diseases interact; they clearly do. Two questions should be separated. First, does CKM meet the criteria of a clinical syndrome: a biologically coherent entity, a predictable pattern of progression, and prognostic value beyond its constituent diseases? Second, is it useful as an integrative framework: promoting coordinated care, guiding therapy, and improving decision-making beyond existing tools? These are different standards, and CKM may succeed as a framework while falling short as a syndrome; a distinction reinforced by the fact that CKM functions simultaneously as a syndrome, a staging system, a risk-stratification construct, and a therapeutic framework, each requiring its own validation (biological coherence, prognostic performance beyond individual components, incremental predictive utility, and demonstrated therapeutic benefit, respectively). The sections that follow are organized accordingly.

## Biological Coherence

Conventionally, a syndrome implies more than the coexistence of related conditions; it suggests a recognizable entity linked by common biological mechanisms. While obesity, diabetes, CKD, heart failure, and atherosclerotic cardiovascular disease (ASCVD) frequently coexist and influence one another, they arise through diverse pathophysiologic pathways and often progress independently. Current CKM studies consistently demonstrate associations between the staging system and biomarkers, cardiac remodeling, inflammation, or adverse outcomes, but association alone does not establish a common biological pathway. Studies have revealed marked biological heterogeneity within individual CKM stages, with distinct proteomic, genetic, and clinical trajectories despite identical stage assignments [3]. The same pattern is evident even within the metabolic risk factors CKM captures: using data-driven cluster analysis, Cho et al. [4] found three subgroups with markedly different cardiovascular risk, even among people who met the same number of metabolic syndrome criteria. Simply counting criteria missed the highest-risk individuals that subtyping identified. If heterogeneity is this pronounced within a single metabolic construct, the broader CKM classification is likely to obscure clinically meaningful biological differences further. Until a unifying pathophysiology is established, CKM remains primarily a descriptive umbrella framework rather than a biologically coherent syndrome.

## Biological Progression

Staging systems are often taken to imply progressive evolution along a relatively predictable continuum, though CKM staging is intended to capture increasing risk and preventive opportunities rather than a mandatory sequence, and stage regression is explicitly recognized. Cancer staging, CKD staging, and heart failure classifications generally reflect increasing severity of a single disease process. CKM staging, by contrast, was deliberately constructed to integrate several interacting disease processes rather than to track one disorder, so the analogy is necessarily imperfect; nonetheless, its stages still combine heterogeneous conditions that do not necessarily represent sequential progression. A patient with obesity may remain metabolically healthy for decades, whereas another patient may develop advanced CKD without obesity or diabetes.

Long et al. [5] reconstructed 3,351 unique disease trajectories from over 1.4 million diagnostic records in the UK Biobank and found that real-world temporal sequences deviate from an orderly stage-wise continuum. Progression was driven by specific disease combinations rather than by advancement through a uniform stage sequence; for example, the markedly greater CKD risk associated with hypertension plus diabetes compared with other hypertensive phenotypes argues for heterogeneous clinical trajectories rather than a predictable stepwise continuum. Consistent with this, Cho et al. [6] found that CKM stage 1 (excess adiposity alone) showed no significant association with cardiovascular risk compared to stage 0 in a cohort of more than 9,000 participants, and that hypertension alone was the dominant driver of all 3 cardiovascular outcomes, again suggesting that the individual component, not the composite stage, determined risk trajectory. These findings are best read as questioning whether the stages represent a uniform natural history, rather than as invalidating the staging construct itself.

## Prognostic Validation

Several studies have reported that higher CKM stages are associated with increased risks of cardiovascular events, kidney failure, and mortality. While these findings have been interpreted as validating the syndrome, they largely reflect how the stages themselves are defined. It is important to distinguish two separate claims. That CKM staging stratifies absolute cardiovascular and kidney risk is expected and, indeed, is part of its stated purpose; higher stages are defined by conditions that themselves confer higher risk. Whether the construct provides incremental prognostic information beyond these constituent conditions is a distinct and more demanding question. Tsai et al. [7] analyzed over 500,000 participants over a median of 16.5 years and reported that CKM syndrome was associated with higher risks of all-cause and cardiovascular mortality. However, when the individual components were analyzed separately, each additional CKM component (hypertension, diabetes, etc.) independently accounted for a 22% increase in mortality, with each component reducing life expectancy by approximately 3 years. This raises the question of whether the prognostic gradient across CKM stages reflects emergent risk beyond what would be expected from the cumulative burden of the individual conditions that were actually used to define those stages.

Moreover, patients within the same CKM stage may have fundamentally different risk trajectories. In the Multi-Ethnic Study of Atherosclerosis (MESA), Ahmad et al. [8] found that the association between CKM stages and cardiovascular outcomes was significantly modified by interleukin-6 levels. Participants with above-median IL-6 at CKM stage 3 experienced a 2.5-year reduction in ASCVD-free survival compared to stage 0, whereas those with below-median IL-6 experienced only a 1.46-year reduction (interaction *p* = 0.002). This within-stage heterogeneity suggests that the current staging system may lack the granularity needed for meaningful prognostic stratification, and that additional phenotyping within stages, rather than abandonment of staging, may be required to capture these distinct risk trajectories. Risk stratification by design should not be conflated with incremental predictive performance. Whether CKM adds prognostic information beyond its individual components (i.e., the more important and still unanswered question) remains the most robust basis for reserving judgment on its status as a distinct syndrome.

## Clinical Utility

Demonstrating statistical associations is an important first step but does not, by itself, establish clinical utility. The fundamental question is not whether CKM stages are associated with adverse outcomes, but whether the framework provides information that meaningfully improves clinical decision-making beyond existing approaches. Clinicians already integrate age, blood pressure, diabetes, kidney function, albuminuria, heart failure, and established cardiovascular disease when assessing risk. As with any proposed clinical syndrome, its incremental value would ideally be demonstrated through improvements in discrimination, calibration, risk reclassification, therapeutic selection, or patient outcomes beyond these established tools.

The AHA’s own 2026 scientific statement on criteria for evaluating novel cardiovascular risk models explicitly requires that any new prediction framework demonstrate incremental predictive utility, including change in C-statistic, categorical net reclassification improvement (NRI), and decision curve analysis, when compared with the standard of care [9]. Yet these very standards have not been applied to the CKM staging system itself. Even the most favorable head-to-head comparison of PREVENT against existing models in two CKD cohorts reported only C-statistic comparisons and found that the improvement in discrimination for cardiovascular events was negligible (ΔC = 0.016 compared with the pooled cohort equations) [10]. NRI, integrated discrimination improvement, and decision curve analysis were not performed, and the apparent superiority of PREVENT for the composite cardio-kidney outcome turned out to be driven predominantly by major adverse kidney events, an expected result of circularity since PREVENT already includes kidney parameters while the comparators largely do not.

Predicted cardiovascular risk is one route into advanced CKM staging, alongside very-high-risk CKD and subclinical cardiovascular disease, and this component depends heavily on the PREVENT equations. Diao et al. [11] applied them to a nationally representative sample of 7,765 US adults and found that they reclassified 53% to lower risk categories compared with the pooled cohort equations, reducing statin eligibility by 14.3 million and antihypertensive eligibility by 2.62 million; changes that the authors estimated could result in 107,000 additional ASCVD events over 10 years. If PREVENT systematically underestimates risk relative to prior models, the thresholds embedded in this component of CKM staging may similarly under-identify high-risk individuals who would benefit from certain therapies; a limitation affecting one qualifying criterion for advanced staging rather than the framework as a whole.

## Inclusivity and Specificity

While inclusiveness is a key strength of the CKM concept, it may also adversely affect its specificity. As additional modifiers (obesity phenotypes, social determinants, imaging, biomarkers, genetic and inflammatory factors) are incorporated, the syndrome's boundaries become increasingly difficult to define. The CKM framework has already begun to expand beyond its original boundaries: the 2026 guideline formally incorporates metabolic dysfunction-associated steatotic liver disease screening and management into CKM care, and recent proposals have advocated renaming the construct cardiovascular-kidney-liver-metabolic syndrome to reflect the addition of hepatic involvement [12, 13]. A syndrome that continually expands to encompass nearly all major chronic diseases risks losing the specificity that gives syndromic classifications their clinical value. Aggarwal et al. [14] applied the CKM staging criteria to more than 10,000 participants from the National Health and Nutrition Examination Survey (NHANES) and found that only 10.6% of US adults were CKM stage 0 (i.e., no risk factors). Such findings certainly emphasize the enormous public health burden of cardiovascular, kidney, and metabolic diseases. A high prevalence is not per se an argument against the construct because the framework intentionally captures individuals at risk. As discussed above, the more relevant question is whether stage assignment adds actionable information beyond its individual components.

## Therapeutic Value

One of the key drivers behind the development of the CKM concept has been the recent emergence of therapies that confer beneficial effects across multiple organ systems, such as sodium-glucose cotransporter 2 (SGLT-2) inhibitors and glucagon-like peptide-1 (GLP-1) receptor agonists. The landmark trials that underpin CKM-directed therapy enrolled patients based on specific diagnoses: type 2 diabetes with established cardiovascular disease, CKD with albuminuria, or obesity with ASCVD [15, 16]. None of these trials recruited participants based on CKM staging, and none tested whether a CKM-guided therapeutic strategy improves outcomes compared with standard disease-specific management. For example, Semaglutide Effects on Cardiovascular Outcomes in People with Overweight or Obesity (SELECT) enrolled adults with BMI ≥27 kg/m^2^ and established ASCVD but excluded those with diabetes, and Finerenone in Reducing Kidney Failure and Disease Progression in Diabetic Kidney Disease (FIDELIO-DKD) enrolled adults with type 2 diabetes and albuminuric CKD; both tested their agents in disease-specific populations rather than CKM-defined cohorts [15, 16]. CKM proponents might argue that the framework is intended to guide which therapies to consider rather than to serve as trial enrollment criteria. However, this distinction underscores the gap: if CKM staging is meant to direct therapy, its ability to do so should be tested against disease-specific management in prospective studies. The observation that agents developed within individual disease domains subsequently demonstrated benefit across cardiovascular, kidney, and metabolic conditions does provide legitimate rationale for considering these diseases together. It does not, however, establish that care organized around CKM staging is superior to optimal disease-specific management. The clinically testable question is, therefore, focused: does organizing and delivering established therapies according to CKM staging improve treatment uptake, therapeutic selection, interdisciplinary coordination, or clinical outcomes compared with high-quality disease-specific guideline-based care? Until this is tested, the therapeutic justification for the syndrome remains an assumption rather than a demonstrated fact.

## Conclusion

The threshold for establishing a new clinical syndrome should remain high. The trajectory of metabolic syndrome, extensively studied yet rarely used as a formal clinical diagnosis, offers a brief but instructive precedent [17]. The advocacy behind CKM is well-founded: cardiovascular, kidney, and metabolic diseases represent an enormous public health burden, and the initiative has meaningfully advanced interdisciplinary collaboration and earlier recognition of shared risk factors. Several of the uncertainties raised here are also acknowledged within the CKM framework itself. It is worth distinguishing two categories: knowledge gaps expected of any evolving framework, which further research will progressively fill, and emerging evidence that specifically challenges assumptions underlying the construct, such as the biological heterogeneity within stages and the absence of demonstrated incremental prognostic value. Our critique concerns the latter primarily. Importantly, the framework’s clinical usefulness need not rest on its status as a distinct biological syndrome: by reducing fragmentation across cardiology, nephrology, endocrinology, and primary care and facilitating uptake of therapies with overlapping cardiovascular and kidney benefits, CKM-based organization of care may offer value on its own terms. The more tractable question is, therefore, not whether CKM qualifies as a syndrome, but whether prospective studies show that organizing care around it delivers measurable advantages in therapeutic uptake, coordination, or outcomes over high-quality disease-specific management. Demonstrating incremental predictive utility over established tools (change in C-statistic, categorical NRI, and decision curve analysis as the AHA’s own 2026 criteria require) would further strengthen the case. Until such data emerge, CKM is best regarded as a promising organizational framework whose value as a distinct clinical syndrome remains to be proven.

## Acknowledgment

ChatGPT (OpenAI) was used for basic language editing, refinement of grammar and style, and formatting of the references.

## Conflict of Interest Statement

Amir Kazory, Abhilash Koratala, and Claudio Ronco were members of the journal’s Editorial Board at the time of submission.

## Funding Sources

No funding has been received for the preparation of this article.

## Author Contributions

A.Kz. crafted the initial draft. A.Ko. and C.R. reviewed and provided critical revisions and additions.
